# Molecular mechanisms of mesoporous silica formation from colloid solution: Ripening-reactions arrest hollow network structures

**DOI:** 10.1371/journal.pone.0212731

**Published:** 2019-03-07

**Authors:** Bahanur Becit, Patrick Duchstein, Dirk Zahn

**Affiliations:** Computer Chemie Centrum, Lehrstuhl für Theoretische Chemie, Friedrich-Alexander Universität Erlangen-Nürnberg, Erlangen, Germany; Institute of Materials Science, GERMANY

## Abstract

The agglomeration of silica nanoparticles in aqueous solution is investigated from molecular simulations. Mimicking destabilization of colloidal solutions by full removal of protective moieties or surface charge, association of SiO_2_/Si(OH)_4_ core/shell particles leads to rapid proton transfer reactions that account for local silanole → silica ripening reactions. Yet, such virtually barrier-less binding is only observed within a limited contact zone. Agglomeration hence leads to the formation of oligomers of nanoparticles, whilst full merging into a compact precipitate is hampered by the need for extended structural reorganisation. Implementing sufficiently fast supply from colloidal solution, our simulations show the development of silica networks comprised of covalently bound, yet not fully merged nanoparticles. Within the oligomerized nanoparticle network, coordination numbers range from 2 to 5 –which is far below closest packing. Our simulations hence rationalize the formation of covalently bound network structures hosting extended pores. The resulting interfaces to the solvent show water immobilization only for the immediate contact layers, whilst the inner pores exhibit solvent mobility akin to bulk water.

## Introduction

In the past few decades, silica particles and nanocomposites thereof were used in many applications ranging from catalysts to coatings and reinforced plastics.[1–4] In addition to this, more recent developments suggest silica nanomaterials for medical and biotechnological applications.[5–7] In bionanotechnological terms, colloidal amorphous silica is taken as a base material to form mesoporous silica. Its large surface area is ideal for hosting molecules, thus empowering mesoporous silica to be used as biocatalysts and drug/gene delivery vehicles, and also for biomimetic processes like bone tissue engineering.[8–12]

Despite such broad interest, our in-depth (i.e. atomic scale) understanding is rather limited. Size-controlled synthesis and functionalizing of silica nanoparticles for customizing properties calls for profound knowledge of the behavior in solution. Understanding and controlling interfacial interactions, association and aggregation is undoubtedly vital to the goal-oriented use of colloidal silica particles.[3] This motivated experimental and computational studies of colloidal silica coagulation, aggregation, adsorption and self-assembly.[13–15] Along those lines, molecular simulation studies appear particularly promising to rationalize atomistic scale processes that are hard to characterize from the experiment.[16] We argue that this perspective will gain even further importance with the increasing complexity of nanomaterials. Indeed, bioglasses call for the rational design of mesoporous silica—which requires understanding pore formation and stability along with the underlying physicochemical properties like cavity percolation, pore shape and dimensions.[17,18]

The aim of the present work is to outline a molecular simulation approach to colloidal silica association, ripening reactions and the formation of mesoporous precipitates. This calls for profound characterization of molecular interactions and the assessment of extended relaxation processes at the same time. To cope with the inherent time-length scale challenge to molecular simulation, we employ the Kawska-Zahn approach originally developed for studying crystal nucleation from solution.[19,20] While mainly relying on efficient molecular mechanics models[21], this approach also allows the consideration of redox-reactions[22] and proton transfer processes[23] to account for ripening reactions occurring during precipitation.

## Models and methods

**Fig 1:**
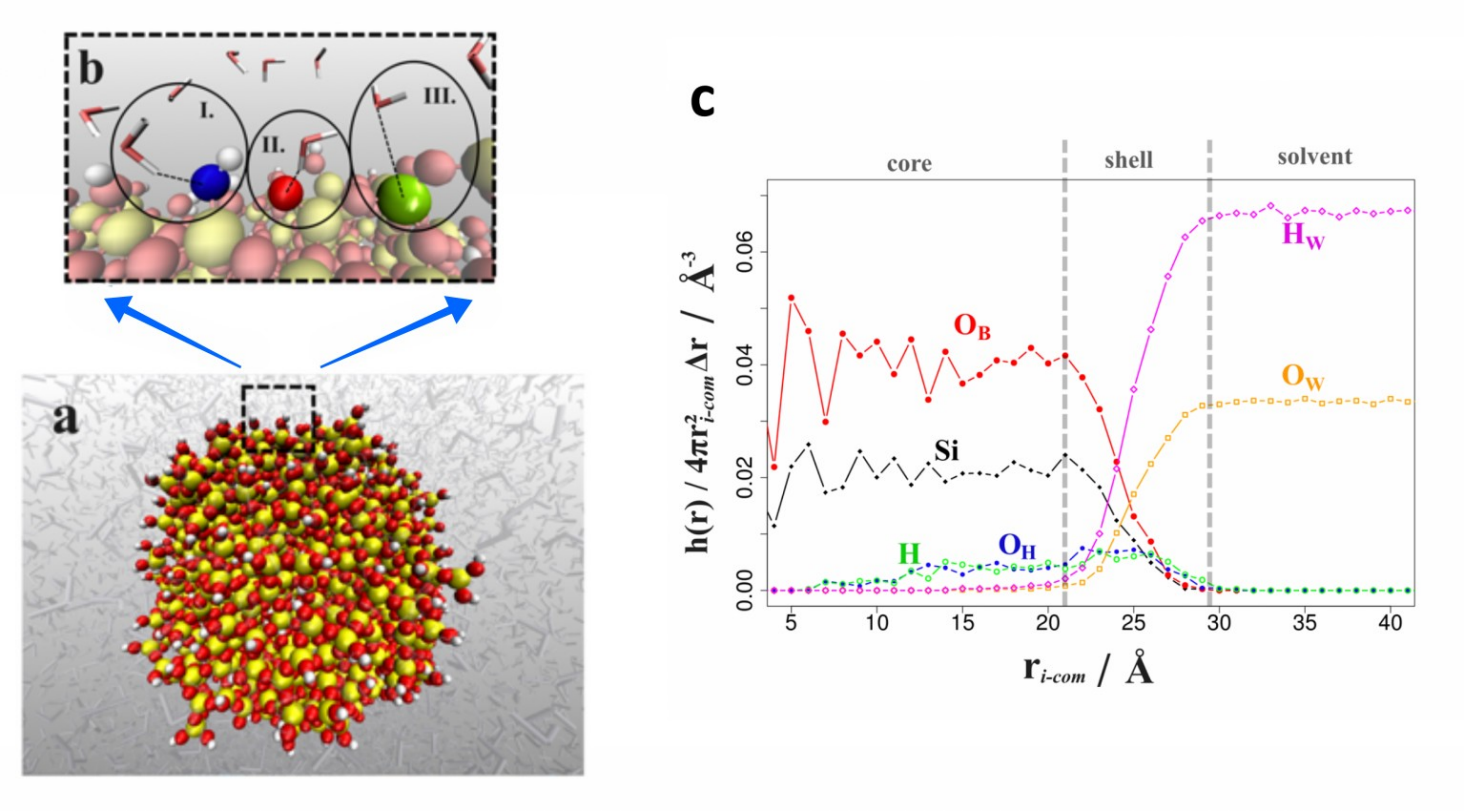
Silica colloid model in aqueous solution. (A) Illustration of the silica colloid model in aqueous solution. (B) Close look at the surface is shown. Hydroxy- (I., O(OH^-^) shown in blue) and oxo-ions (II., O^2-^ highlighted in dark red) act as acceptors to hydrogen bonding with water molecules, while the silicon ions form salt-bridges to the oxygen atoms of nearby water molecules (III., Si colored green). (C) Normalized radial density profiles as functions of the distance from the colloid center, indicating a SiO_2_ core zone, a SiO_x_(OH)_4-2x_ shell area and the solvent interface, respectively. We note that there is a small, yet non-zero density of hydroxide ions in the particle interior. While this feature is indeed also observed from experiments [1], a quantitative assessment would call for more rigorous configuration sampling. Atom colors: Si(yellow), O(red), H(white).

To create a silica colloid model, SiO_2_/Si(OH)_4_ core/shell particles were prepared from a nanoblock (~cube of 38.5 Å dimensions) of alpha-quartz comprising 8 ×8×7 unit cells. Therein, the surface oxygen atoms were alternatively depleted / protonated. Using molecular dynamics simulations in vacuum, amorphous colloids were obtained from melting (imposing 2300 K) and rapid cooling to 300 K within 0.5 ns. The stability of the resulting nanosphere of ~ 5 nm diameter (Fig 1A) was then confirmed from additional 3 ns runs at room temperature. While this procedure may be applied to generating nano-spheres of a large variety of sizes, the dimensions chosen for the present work were motivated from the experimental studies reported in refs. [5–12].

With the exception of proton transfer reactions, all interactions are described by molecular mechanics models adopted from the literature[24–26] and reported in detail in the supplementary information (i.e. S1 File). Namely, the CMAS94 model from Matsui et al. was chosen for the Si-O^2-^ interactions [24]. This is combined with the tailor-made potentials from Ciacchi et. al. [26] and the TIP3Pm water model of Mark et. al. [25] to model silica-water interface interactions. Validation of the models is provided in the results part in terms of structural properties of the nano-sphere particle and of the particle-water interface.

To account for the cascade of possible proton transfer reactions occurring in the course of silica aggregation, we use an inexpensive quantum/molecular mechanics scheme to discriminate exothermic from unfavorable ripening reactions.[23] The OH^-^∙∙OH^-^ hydrogen bonds that candidate for proton transfer were selected on a H∙∙O distance threshold of 3.0 Å (chosen generously large to avoid missing possible reaction partners). For each possible ripening reaction, relaxed structures and energy levels of the reactant and product states are sampled from molecular dynamics runs using force-fields[24,26]. The average difference in energy ΔE^MM^ (full model) is corrected for the different proton affinity as derived from quantum calculations of the reacting hydroxide ions. Hence, the energy change upon ripening is estimated from Eq 1.

$$\Delta E_{QM/MM}=\Delta E_{MM}\left(\mathrm{full}\;\mathrm{model}\right)+\Delta E_{QM}\left(\mathrm{subsystem}\right)-\Delta E_{MM}\left(\mathrm{subsystem}\right)$$ (1)

The underlying subsystem should be chosen as small as possible to provide the computational efficiency required to scan hundreds to thousands of possible ripening reaction steps. In an earlier study of ZnO formation from zinc and hydroxy ions, proton transfer could be described by considering only the two reacting OH^-^ ions quantum mechanically.[23] For the present silica/silanol system, we found that charge transfer at least from the O^2-^ to its nearest metal ion must be taken into account. Hence, the quantum mechanically treated subsystem was taken as [HO-Si-OH]^2+^ → [SiO]^2+^ + H_2_O in the gas phase. The error margins of this approach may be estimated by shifting further neighbors from the MM to the QM treatment. This was found to change Δ*E*_*QM/MM*_ by several tens of kJ/mol (whilst the absolute values are in the order of 200–500 kJ/mol). Our QM/MM calculations hence aim at fast discrimination of exothermic/endothermic proton transfer attempts based on rough estimates with up to 20% error margins.

**Fig 2:**
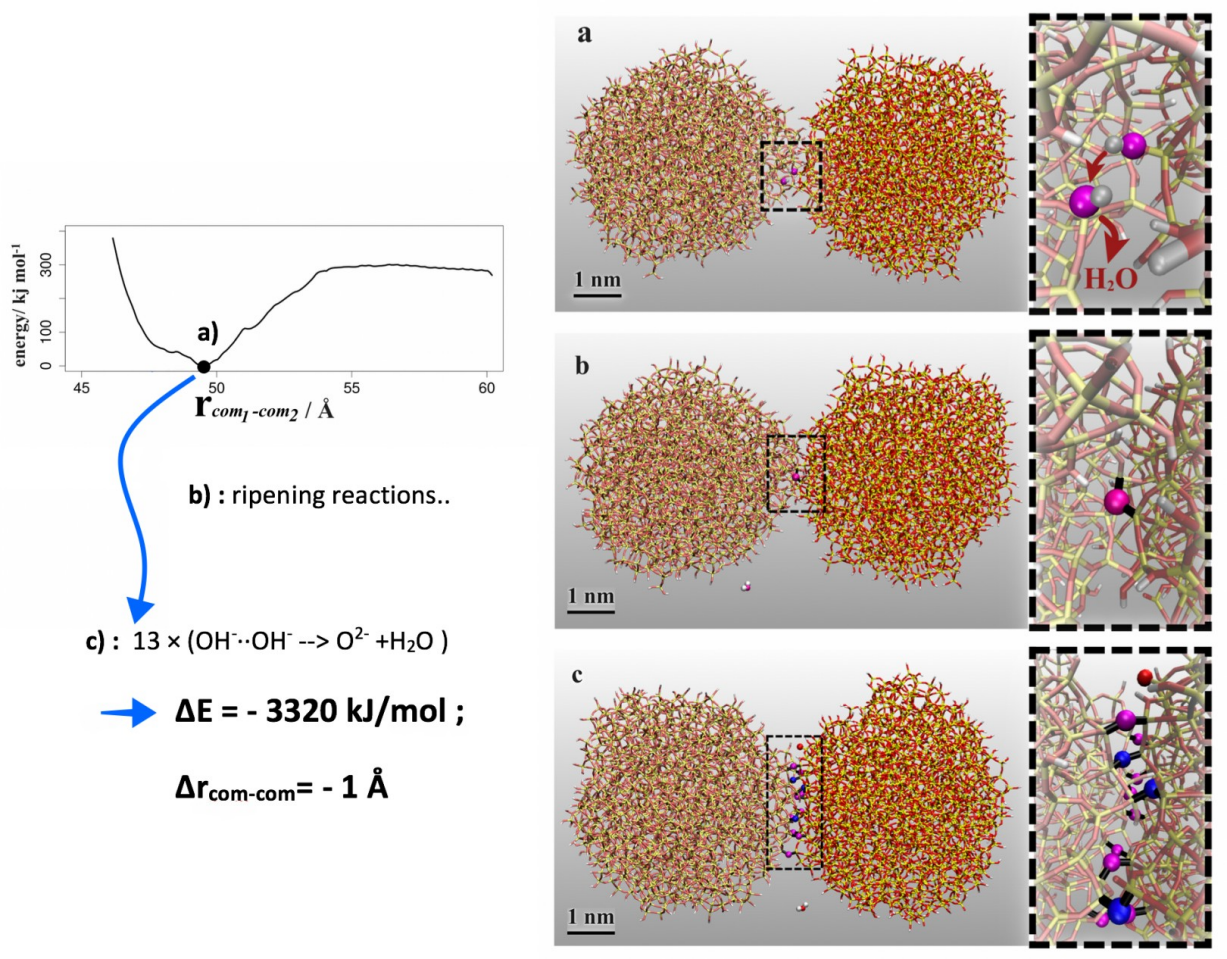
Potential-of-mean force profile for the approaching of two silica particles and colloidal contact by hydrogen bonding and salt-bridges and dimer formation. Ripening reactions and structural relaxation (a) → (b) then lead to exothermic formation of O^2-^ ions that bridge the two colloids and (c) finally lead to a peanut-shaped dimer. In each of these ripening events, the resulting water molecule is migrated into the solution (omitted for clarity in snapshots a-c). Atom colors are as in Fig 1, but highlighting the O^2-^ ions formed by ripening reactions. The newly formed oxo-ions establish Si-O-Si bridges between the two colloids, while partly maintaining the previous O-Si contacts (O^2-^ colored magenta), or by rearranging its entire coordination structure. (O^2-^ colored blue).

The approaching of two silica colloids in water was first investigated from potential of mean force calculations using restraints on the colloid center-of-mass distances (from 6 to 4.6 nm in steps of 0.01 nm, each sampled from 100 ps runs) to characterize the permeation of solvent shells and particle contact, respectively.[27] For the assessment of larger agglomerates, we iteratively model particle-by-particle association and relaxation using the Kawska-Zahn simulation scheme.[19,23] Along those lines, solute diffusion to a forming aggregate is mimicked by a simple docking approach to save computational costs. Based on random incoming directions, particle association is boosted by temporarily removing the solvent. While keeping the forming aggregate fixed, the newly incoming particle is placed at a 5.4 nm distance which reflects the onset of the attractive force observed from the potential of mean force calculations (Fig 2). After simple steepest descent minimization, full relaxation of the docked state, including possible ripening reactions, is performed from 10 ns scale simulation runs in aqueous solution.[19,23]

All MD simulations in this study were performed with LAMMPS using a time step of 1 fs.[28] Aqueous solution was mimicked by periodic boundary conditions applied to cubic simulation cells of 15 nm to 30 nm dimensions, depending on the size of the solvated silica agglomerate. Relaxation at 300 K and 1 atm was performed by the Nose/Hoover thermostat/barostat combination using damping constants of 0.1 ps and 1 ps, respectively. To account for long-range interactions, damped shifted force potentials with a cut-off radius of 1.2 nm were applied.[29,30] The quantum calculations were performed at the MP2/6-311+G(d,p) level using Gaussian 09.[31]

## Results

The silica colloid model was first subjected to a structural analysis to ensure reasonable starting points before investigating particle association and the agglomeration of precipitates. Upon melting and re-solidification of the core-shell quartz nano-cube, we obtained spherical silica colloids of amorphous inner SiO_2_ core, whilst the surface comprises of silanol groups. The distribution of Si^4+^, O^2-^ and OH^-^ ions as functions of the distance from the center-of-mass is shown in Fig 1. We find the corresponding density profiles in line with the comparable simulation studies of Wendland[32] and Singer[33]. This also holds for the interactions with water molecules, once the colloid is immersed into aqueous solution. Interfacial water molecules form O(water)∙∙Si salt-bridges and H(water)∙∙O(OH^-^ and O^2-^) hydrogen bonds with the silica particle. While it is intuitive to expect such strong electrostatic interactions to provoke well-defined solvation shells, the local roughness of the particle (as reflected by the overlap of water and ion density profiles of almost 1 nm) prevents the formation of spherical solvent shells (Fig 1).

**Fig 3:**
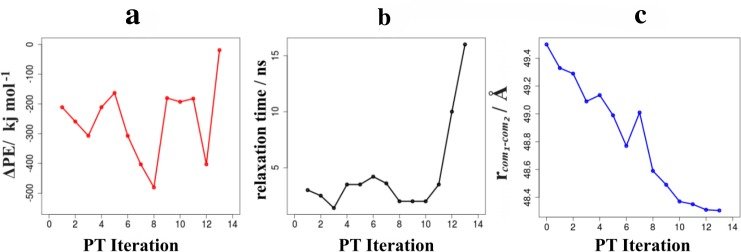
Characterization of the proton transfer (PT) events and structural relaxation of the colloid-colloid contact into a dimer. (A) Energy change upon ripening (B) Relaxation time required and (C) resulting colloid-colloid distance as functions of the individual PT steps. Convergence is indicated by the reaction energy (endothermic for any candidate of PT iterations beyond 13), the raise in required time scales for structural relaxation and convergence of the colloid-colloid distance, respectively. We note that the inexpensive QM/MM approach employed for estimating the proton transfer energy is subjected to error margins of tens of kJ/mol. On this basis, the proton transfers 1–12 are clearly identified as exothermic, whilst we caution that the 13^th^ reaction step might already be slightly endothermic.

Experimental setups of colloidal solutions take use of protective groups (such as ethylenoxide for silica prepared from tetraethyl orthosilicate precursors via the Stöber process) surfactants or surface charge to avoid agglomeration.[1,2] In turn, for the syntheses of meso-porous silica, aggregation is induced by removal of the protective moieties or application of electrolytes to neutralize surface charge.[1,2,8] The colloids modelled in our simulations are prepared as stabilizer-free and charge-neutral, and hence mimic silica particles *after* destabilization of the colloidal suspension. Indeed, probing the association of two silica particles (using replicas of our colloid model) in water, we find barrier-less binding of the two colloids. Fig 2 (left) indicates considerable “non-reactive” binding via hydrogen bonds and salt-bridges by ~ 300 kJ/mol. At the colloid-colloid interface we however observed OH^-^∙∙OH^-^ hydrogen bonds that may be subject to proton transfer reactions. Indeed, a total of 13 ripening reactions by exothermic proton transfer and structural relaxation could be observed (Fig 2, right). Within an iterative series, starting with the nearest candidate reactants, both reactant and product states were relaxed in aqueous solution and proton transfer is only implemented if found exothermic (typically by -200 to -400 kJ/mol, see also Fig 3A). Convergence of the resulting structures is monitored by following the system energy as a function of time. The required relaxation time was found in the range of nanoseconds, with the exception of the final ripening event which structural rearrangement was observed as one order of magnitude slower (Fig 3B). In line with annealing of the colloid-colloid binding, the distance of the respective center-of-masses converges to about 1 Å less than the distance of the initial contact pair (Fig 3C). This cascade of proton transfer and structural relaxation steps leads to a covalently bound colloid dimer which is grown together by a well-defined contact zone. Further ripening, and like this eventual merging into a single colloid, is hindered by the absence of further OH^-^∙∙OH^-^ contacts. In other terms, the dimer structure illustrated in Fig 2C is suggested as a long-lived intermediate of colloid association.

Depending on colloid concentration in the solution, the association of further colloids will hence outperform the kinetics of ripening/merging beyond the colloid-colloid bonds described above. Experimentally, the underlying kinetics may be balanced by the choice of solvent, colloid concentration and by surfactant molecules to temporarily hinder the ripening of silanol groups.[17,34,35] Within our simulation protocols, we mimic colloid precipitation at optimal balance of association and ripening kinetics for the formation of mesoporous silica. The association of further silica particles to the aforementioned colloid dimer is implemented by the Kawska-Zahn approach. [23] Thus, colloid association to a forming aggregate is implemented from random incoming directions as a one-by-one docking and relaxation procedure. Therein, agglomerate ripening is considered analogous to the dimer model. We hence model colloid association from a solution reservoir and structural relaxation after docking and ripening of the silanol groups within the newly formed contact zone, only. Thus, ripening is only implemented for the (exothermic) proton transfer events which were observed as immediately accessible.

**Fig 4:**
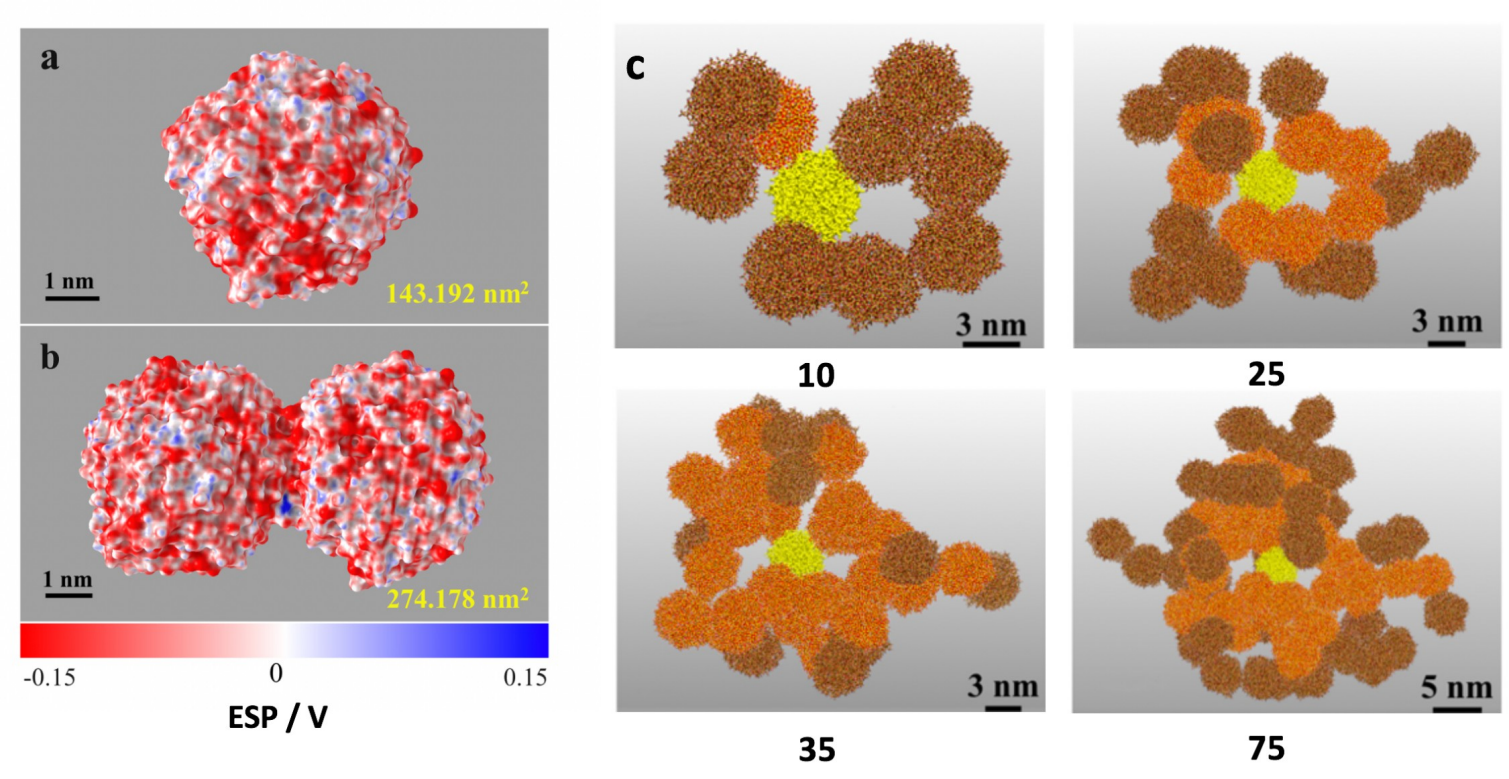
Scale-up modelling of colloid aggregation and ripening into mesoporous silica. Left: illustration of the molecular surface (using the vmdICE plugin[38]) and the local electrostatic potential (ESP) of (A) the colloid model and. (B) the ripened dimer. The contact area is obtained as A_contact_ = A_colloid_—½∙A_dimer_ = 12.2 nm^2^. (C) Snapshots of the agglomeration simulation runs up to association and ripening of 75 colloids. The first colloid is highlighted in yellow, whilst subsequent snapshots use different colors to show old and new building blocks upon 2→10, 10→25, 25→35 and 35→75 evolution of agglomerates are shown in orange and brown, respectively.

In order to achieve a large number of such aggregation processes at reasonable computational costs, in what follows we mimic the (elsewise rather extreme number of) ripening reactions in an approximate manner. For this, the observed number of 13 ripening reaction steps is related to the contact area of the ripened dimer (taken as the reduction in surface area as compared to two separate colloids, Fig 4). Upon docking further colloids, we directly implement the respective proton transfer reactions based on the nearest OH^-^∙∙OH^-^ hydrogen bonds. Therein the overall number of reactive events is interpolated according to:

$$N_{ripening\;steps}=N_{ripening\;steps}\left(\mathrm{colloid}\;\mathrm{dimer}\right)\times \frac{A_{contact}}{A_{contact}\left(\mathrm{colloid}\;\mathrm{dimer}\right)}$$ (2)

On this basis, the association and ripening of agglomerates comprising up to 75 colloids could be achieved (Fig 4). The resulting aggregates extend over several tens of nanometers which suffices to assess bulk domains comprised of a percolating network of inter-grown colloids separated by nm-scale pores. With the atomic models at hand, we can now perform a series of analyses to illustrate the in-depth characterization enabled by the multi-scale models.

**Fig 5:**
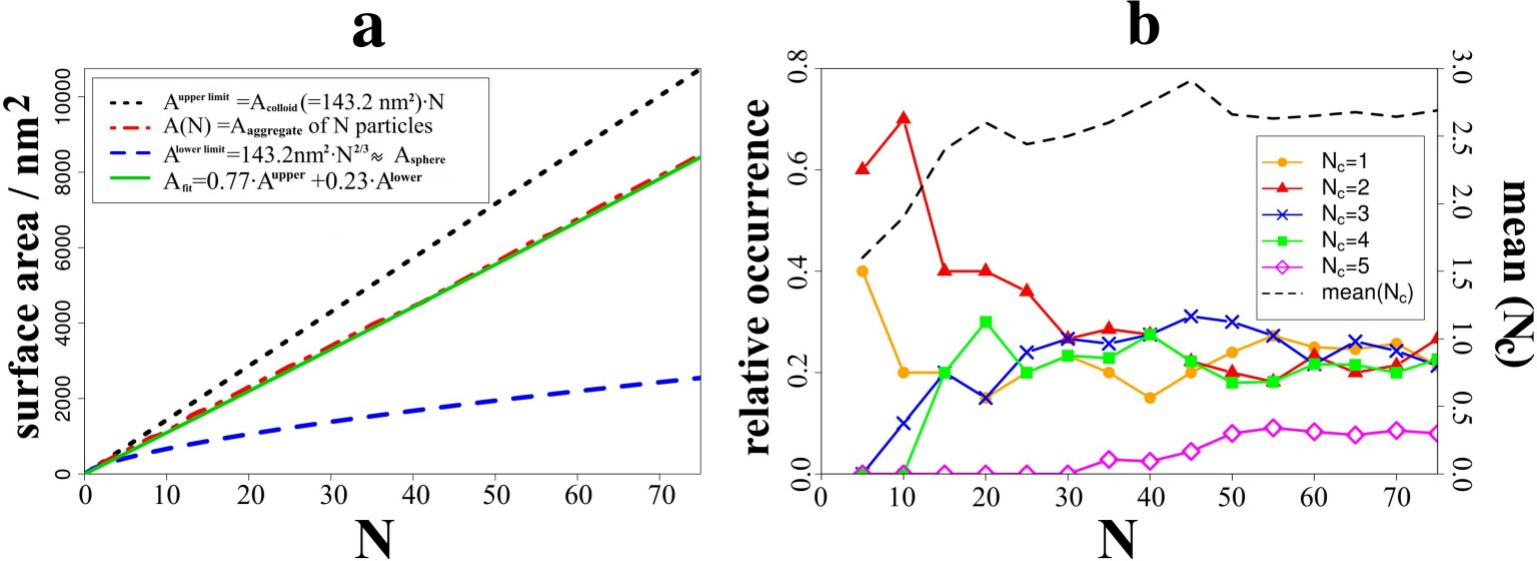
Evolution of the surface area and the occurrence of structural motifs as functions of agglomerate size. (A) The degree of porosity may be illustrated by comparing the molecular surface area of the agglomerate to the theoretical boundaries of the total surface area of fully dispersed colloids (77% agreement) and merging into a single sphere (23% match). (B) Occurrence profiles of the coordination numbers for the colloidal building blocks, normalized with respect to the total number of colloids in the aggregate.

To quantify the porosity of the resulting structures, we calculated the molecular surface of the silica agglomerate (i.e. the surface area accessible to solvent molecules) as a function of aggregate size N–with N being the number of colloids grown together (Fig 5, left). It is educative to compare the surface area of the porous agglomerate A(N) to that of N dispersed colloids before association and to the putative surface area of a larger silica sphere as would be obtained from full merging of N colloids. In other terms, N×A(1) reflects the *upper limit* of the surface area possible for the porous aggregate, whilst the *lower limit* is estimated from an idealized merged sphere model with N times the colloid volume (and thus N^2/3^ times the surface area):

N×Acolloid=N×Aaggregate(1)≥Aaggregate(N)≥Amergedsphere=Acolloid×N23 (3)

On this scale, the degree of porosity can be ‘quantified’ in terms of mixing the limiting functions to match the observed profile of A(N) (see also Fig 5A). For the porous silica models obtained from our aggregation procedure (Fig 4) we find the surface area as much as 77% of the theoretical maximum given in Eq 3.

The origin to this high level of porosity is the connectivity of the colloids forming its building blocks. We therefore performed a coordination number analysis of colloid-colloid contacts as a function of aggregate size (Fig 5B). While it is tricky to clearly discriminate surface and bulk domains in a porous aggregate, we suggest the number of surface- and bulk-type motifs should nevertheless evolve with N^2/3^ and N, respectively.[36] Because of normalization by 1/N, the size-dependent profiles of coordination number occurrences hence allow distinguishing colloid coordination in the bulk (converges to constant ratios) and the aggregate surface (motif occurrences scale with N^-1/3^). While a proper quantitative assessment would call for larger aggregates, our simulation runs at least allow a qualitative identification of motif occurrence in the bulk as predominantly N_c_ = 2,3 and 4 –whilst a minor fraction of N_c_ = 5 is observed. To this end, we can rationalize the way that pores result from loops of connected colloids. We find 2, 3 and 4-fold coordination at roughly equal occurrence. While the former coordination numbers (2,3) would call for 2-dimensional aggregates, 3-D structures stem from N_c_ ≥ 4. We hence find a (roughly) 2:1 blend of 2D and 3D type connectivity for the colloidal building blocks that explains the formation of loops from filling 3-dimensional space by a large fraction of 2-dimensional motifs.

**Fig 6:**
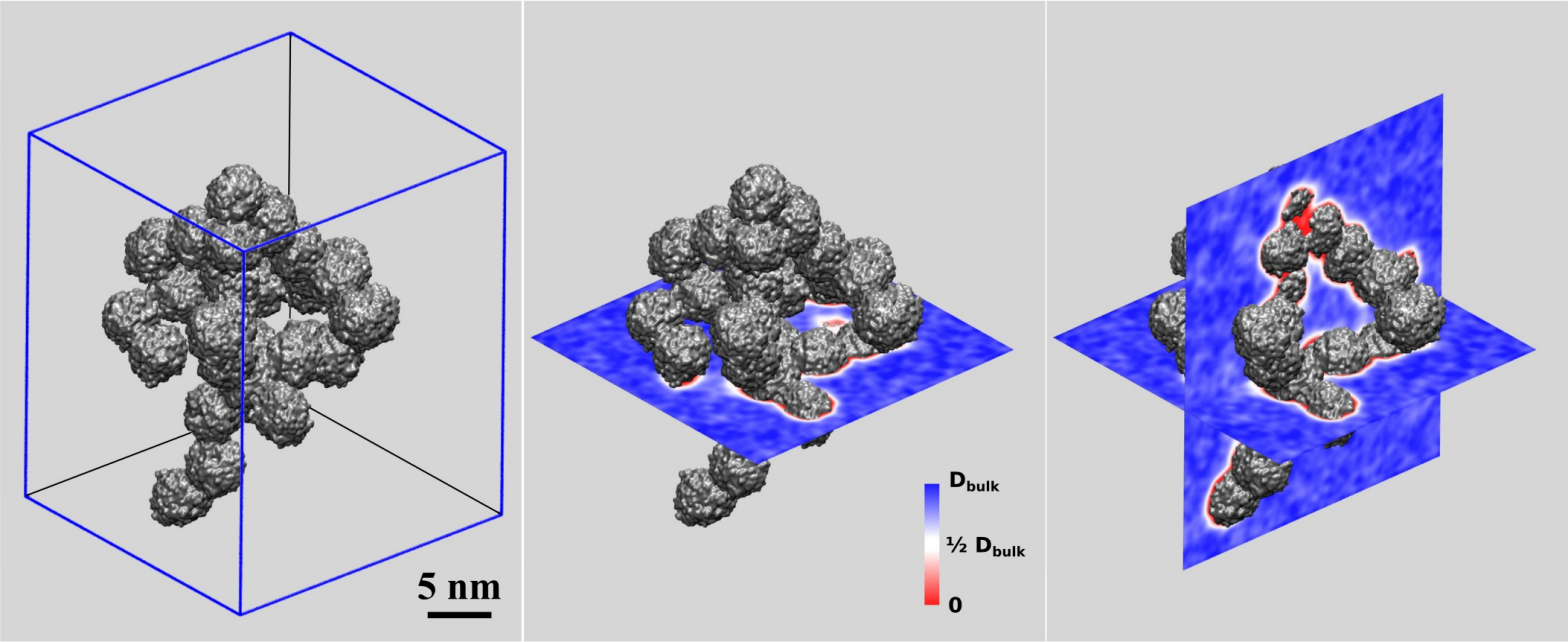
Long-scale characterization (6 ns) of water mobility next to the silica aggregate comprising 35 colloids. Using periodic boundary conditions, aggregate solvation in physiologic NaCl solution is mimicked by a cubic cell comprising ~ 1 million water molecules. While the diffusion constants of water molecules at the immediate contact to silica are observed as 0.1–0.5 of the bulk value, the pore dimensions are found sufficiently large to permit nm-scale domains of bulk-like solvent mobility.

To characterize the interplay of the meso-porous silica precipitate with penetrating solvent, we performed a large scale (6 ns) molecular dynamics run of the aggregate comprising 35 colloids (see also Fig 4C). The solvent was chosen as a NaCl—water solution according to physiologic salt concentration (c[NaCl] = 0.15 mol /L). This allows assessing the behavior of both water and ions at the interface to the silica surfaces. For both Na^+^ and Cl^-^ we find poor association to the silica surface. The interface to silica is hence almost exclusively formed by water molecules which in turn experience significant reduction in mobility. This applies to two layers of solvation which in total extend over approximately 0.8 nm from the surface of particle. For quantification of the latter issue, we computed a 3-dimensional profile of ‘local’ water diffusion constants, by dividing space into a 0.2 nm sized grid and sampling the mean-squared displacement of water molecules for each of the grid points.[37] The resulting mobility profile is illustrated in Fig 6. While being an exemplary case study, our mobility analysis demonstrates that pore dimensions beyond 2 nm are required to allow permeation of water at bulk-like diffusion constants. For the present aggregate based on 5 nm sized colloid building blocks, the pores clearly extend this threshold. Indeed, the pore dimensions range from 3–8 nm, hence allowing the exchange of water and Na^+^/Cl^-^ ions between the meso-porous interior and the embedding solution, respectively. It is intuitive to expect similar permeation also for small solute molecules–as suggested for pharmaceutics release from meso-porous silica vehicles.[5–7]

## Conclusion

Molecular simulations may bridge the scales from reacting ions to the agglomeration of nanoparticles leading to (largely) unprejudiced models of complex nanomaterials. Starting from individual association events and ripening reactions our simulation protocols unravel the nature of cavities and pores formation and provide atomic-scale details of surface/interface properties. Silica surely reflects a particularly prominent case study, however the modelling techniques are widely transferrable to characterizing meso-porous materials based on building-block association.

While the present work reveals the foundations of porosity and solvent permeation, the resulting models paved the way to a large variety of future studies. These may involve the role of surfactants to control silica association and the use of colloids with disperse size distribution.[17] Apart from syntheses, the perspectives also include the characterization of meso-porous silica in terms of releasing guest molecules and ion impregnation from solution to rationalize application as vehicles for drug delivery and as bio-glass materials, respectively.

The latter perspective calls for much larger aggregates, and even 3D-periodic models of mesoporous silica. While impractical to provide from nano-particle by nano-particle association and ripening, we can still employ the beforehand discussed findings to create coarse-grained models that bridge the required time- and length scales. In ongoing efforts, we also introduce an account of additive molecules and their ability to form micelles that stabilize hollow structures as widely used in real syntheses of meso-porous silica. [7,8,10,17]

## Supporting information

S1 File Applied force field parameters for the simulations. (Table A) Van-der-Waals Interaction parameters. (Table B) Partial charges and Lennard-Jones parameters ion-water and water-water interactions.
(DOCX)

S2 File Simulation_Models.zip. Obtained Models of solvated single particle, solvated dimer particle and mesoporous particle.
(ZIP)
