# Supplementary material for: Molecular mechanisms of mesoporous silica formation from colloid solution: Ripening-reactions arrest hollow network structures
Source: PLoS One. 2019 Mar 7;14(3):e0212731. doi: 10.1371/journal.pone.0212731 (PMC6405164; doi:10.1371/journal.pone.0212731)
Supplement: S1 File — (Table A) Van-der-Waals Interaction parameters. (Table B) Partial charges and Lennard-Jones parameters ion-water and water-water interactions. (DOCX) [file pone.0212731.s001.docx]

**Supporting Information**

For the ion-ion interactions of silica, we employed CMAS94 potential developed by Matsui *et. al.* [s1] which is implemented in LAMMPS as

$$V_{ij}=\frac{q_{i}q_{j}}{4\pi\epsilon_{0} r}+{A_{ij}e}^{-r/{}_{ij}}-\frac{C_{ij}}{r^{6}} r<r_{c} (1)$$

where r_c_is cutoff distance.

| *i-j* | A_ij_/ kj mol^-1^ | ρ_ij_/ Å | C_ij_/ Å^6^ kJ mol^-1^ |
| --- | --- | --- | --- |
| Si-Si | 7.7127273x10^12^ | 0.046 | 2430.49 |
| Si- O_B_ | 4843342.1660 | 0.161 | 4467.073 |
| Si- O_H_ | 4843342.1660 | 0.161 | 4467.073 |
| O_B_-O_B_ | 623552.77 | 0.276 | 8210.1712 |
| O_H_- O_H_ | 623552.77 | 0.276 | 8210.1712 |
| O_B-_ O_H_ | 623552.77 | 0. 276 | 8210.1712 |

**Table A. Van-der-Waals Interaction parameters.** Parameters are for ion-ion interactions of silica using the notation O_B_ for O^2-^ and O_H_ for the oxygen atoms of OH^-^ ions, respectively.

All other van-der-Waals interactions (involving water, Na^+^,Cl^-^ and the hydrogen atoms of OH^-^) were described by 12/6 Lennard-Jones potentials, hence (2):

$$V_{ij}=\frac{q_{i}q_{j}}{4\pi\epsilon_{0} r}+4\varepsilon_{ij}\left[ \left( \frac{\sigma_{ij}}{r} \right)^{12}-\left( \frac{\sigma_{ij}}{r} \right)^{6} \right] r<r_{c} (2)$$

where r_c_is cutoff distance.

|  | q / e | σ / Å | ε/kj mol^-1^ |
| --- | --- | --- | --- |
| Si | 1.890 | 2.850876 | 1.2552 |
| O_B_ | -0.945 | 3.139528 | 1.09202 |
| O_H_ | -0.61425 | 2.939966 | 0.6276 |
| H | 0.14175 | 1.7818 | 0.087864 |
| H_W_ | 0.417 | 0 | 0.192464 |
| O_W_ | -0.834 | 3.15066 | 0.6363864 |
| Na | 1.0 | 2.4299 | 0.19623 |
| Cl | -1.0 | 4.4776 | 0.14891 |

**Table B. Partial charges and Lennard-Jones parameters ion-water and water-water interactions**. Parameters are adopted from Ciacchi et al [s2]. The subscript W refers to the water molecules(TIP3Pm, model [s3]).

To account for long-range Coulombic interactions, we mimicked Ewald summation by the damped shifted force approach[s4]:

$$V_{ij}^{Coulomb}=\frac{q_{i}q_{j}}{4\pi\epsilon_{0}}\left[ \frac{erfc\left( \alpha r \right)}{r}-\frac{erfc\left( \alpha r_{c} \right)}{r_{c}}+\left( \frac{erfc\left( \alpha r_{c} \right)}{r_{c}^{2}}+\frac{2\alpha}{\sqrt{\pi}}\frac{\exp\left( -\alpha^{2}r_{c}^{2} \right)}{r_{c}} \right)\left( r-r_{c} \right) \right] r<r_{c}(3)$$

where r_c_is cutoff distance, α= 0.05 Å^-1^the damping parameter and erfc() the complementary error function.

**Supporting References**

s1. Matsui M. A transferable interatomic potential model for crystals and melts in the system. Mineral Mag. 1994;58A: 571–572

s2. Cole DJ, Payne MC, Csányi G, Spearing SM, Ciacchi LC. Development of a classical force field for the oxidized Si surface: Application to hydrophilic wafer bonding. J Chem Phys. 2007;127: 204704. doi:10.1063/1.2799196

s3. Mark P, Nilsson L. Structure and Dynamics of the TIP3P, SPC, and SPC/E Water Models at 298 K. J Phys Chem A. American Chemical Society; 2001;105: 9954–9960. doi:10.1021/jp003020w

s4. Fennell CJ, Gezelter JD. Is the Ewald summation still necessary? Pairwise alternatives to the accepted standard for long-range electrostatics. J Chem Phys. 2006;124: 234104. doi:10.1063/1.2206581
